# WG-FuseNet: Wavelet-Guided and Gated Fusion Network for Road Segmentation

**DOI:** 10.3390/s26010218

**Published:** 2025-12-29

**Authors:** Yu Nie, Jiaqi Sun, Ming Zhu, Yuan Liu, Yuanfu Yuan, Shuhui Jiang, Yan Lu, Jiarong Wang

**Affiliations:** 1 Changchun Institute of Optics, Fine Mechanics and Physics, Chinese Academy of Sciences, Changchun 130033, China; nieyu23@mails.ucas.ac.cn (Y.N.); yuanyuanfu24@mails.ucas.ac.cn (Y.Y.);; 2 University of Chinese Academy of Sciences, Beijing 100049, China; 3 Unit 94259 of the Chinese People’s Liberation Army, Xi’an 710049, China

**Keywords:** road segmentation, wavelet transformation, gated fusion, feature enhancement

## Abstract

In current road segmentation tasks, high-frequency details of roads (such as road edges and pavement textures) tend to become blurred or even lost during feature extraction due to progressive downsampling, leading to imprecise segmentation boundaries. Moreover, existing fusion methods predominantly rely on simple concatenation or summation operations, which struggle to adaptively integrate the rich texture information from RGB modality with the geometric structural information from Depth modality, thereby limiting fusion efficiency. To address these issues, this paper proposes an innovative model. We design a Cross-scale Wavelet Enhancement Module (CWEM) to compensate for the shortcomings of traditional networks in frequency domain analysis, explicitly enhancing the representation capability of edge and texture features. Simultaneously, a Gated Cross-Modality Fusion module (GCMF) is constructed to achieve adaptive and efficient fusion between RGB and Depth features. Additionally, to tackle the high false detection rates and confusion between sidewalks and opposite lanes in existing methods, this paper optimizes the loss function to further improve the model’s discriminative ability in complex scenarios. Experiments on the public KITTI_Road dataset demonstrate that the proposed method achieves a segmentation accuracy of 97.31% while maintaining a real-time inference speed of 34 FPS, with particularly outstanding performance in road edge integrity and shadow area handling.

## 1. Introduction

The rapid advancement of autonomous driving technologies has imposed rigorous demands on environmental perception systems, specifically concerning accuracy, robustness, and real-time performance. As a fundamental task within the perception pipeline, road segmentation aims to precisely delineate drivable regions from obstacles at a pixel-wise level. The fidelity of its output directly governs the decision-making reliability of downstream path planning and motion control, serving as a cornerstone for ensuring the functional safety of autonomous vehicle systems.

Contemporary technical approaches are primarily divided into two categories: monocular vision-based (RGB) methods and multi-sensor fusion-based methods. While deep learning-driven vision-only schemes [1,2] have achieved near-human performance in well-lit, standardized daytime environments, they inherently lack the depth perception of the 3D scene. Consequently, these methods often experience significant performance degradation under adverse conditions, such as intense lighting fluctuations, backlighting, low-light nocturnality, and inclement weather (rain, snow, or fog). This manifests as blurred road boundaries, misclassification of shaded regions, and missed detections of distant obstacles [3], severely constraining their reliable deployment in complex scenarios.

To surmount the limitations inherent in vision-only paradigms, the integration of multi-source sensor information has become a prevailing trend for enhancing the robustness of perception systems. Specifically, LiDAR-based fusion strategies [4] provide precise 3D point cloud data, thereby effectively augmenting the model’s comprehension of scene geometry. However, the sparse and unstructured nature of point clouds imposes a substantial computational burden, making it difficult to satisfy the stringent inference efficiency constraints of onboard embedded platforms [5]. Furthermore, performance degradation in adverse weather conditions (e.g., rain and fog), coupled with high hardware costs, limits the potential for the large-scale deployment of LiDAR.

**Figure 1:**
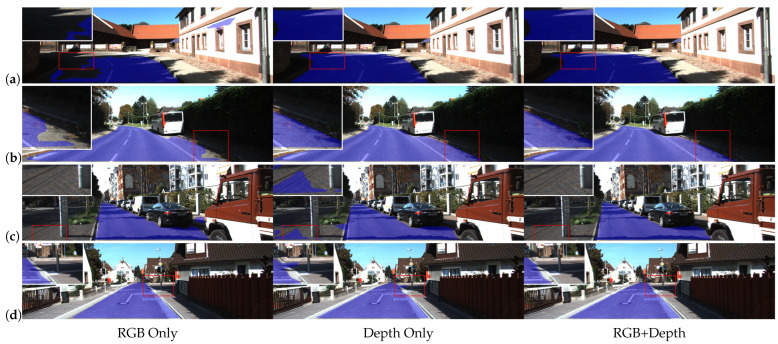
Road segmentation results comparing different modalities. (**a**,**b**) demonstrate the limitations of RGB-only methods when dealing with adverse lighting conditions, such as shadows. (**c**,**d**) illustrate the shortcomings of depth-only data in distinguishing regions with similar depth values. The blue areas denote the predicted road surface, while the red dashed circles highlight segmentation errors.

Figure 1 highlights the inherent limitations of uni-modal approaches. Specifically, (a) and (b) fail to correctly classify road pixels. This failure is attributed to the susceptibility of RGB images to visual artifacts, such as road shadows.Conversely, (c) and (d) erroneously classify non-road regions as drivable areas. This misclassification stems from the geometric ambiguity where these regions are coplanar with the road surface. In contrast, RGB-D-based fusion strategies offer a more pragmatic trade-off between efficiency and performance. By encoding scene geometry into a structured 2D matrix format, depth maps facilitate efficient alignment and fusion with RGB images at the feature level. However, most existing methods merely adapt traditional Convolutional Neural Networks—originally designed for RGB processing—to extract depth features. Consequently, they fail to fully exploit the unique geometric structural information inherent in depth maps, particularly high-frequency edge and contour features. This limitation leads to suboptimal perception of critical details such as road boundaries, curbs, and low-lying obstacles. Furthermore, the lack of in-depth exploration into the complementarity of multi-modal information during the fusion process hinders further improvements in segmentation accuracy and generalization capability.

To address the aforementioned challenges, this paper proposes a Wavelet-Transform-Enhanced RGB-D Road Segmentation Network. By incorporating frequency domain analysis and adaptive fusion mechanisms, our approach aims to bolster the model’s semantic understanding and structural perception capabilities in complex scenarios. The main contributions of this work are summarized as follows:

1. Multi-scale Wavelet Enhancement Module: Leveraging the superior frequency localization properties of the wavelet transform, this module explicitly extracts and amplifies high-frequency edge and detail components from depth maps across multiple scales. Consequently, it significantly enhances the perception of road structural features, thereby mitigating boundary blurring and the loss of fine details;
2. Cross-modal Gated Fusion Module: We design a bi-directional gating mechanism to adaptively select and integrate features from RGB and depth modalities. This mechanism effectively suppresses inter-modal redundancy while emphasizing complementary information, thus elevating the quality of the fused feature representation;
3. Improved Binary Classification Loss: We refine the loss function by introducing a focusing mechanism tailored for hard samples, such as shadowed regions, boundary pixels, and small obstacles. By adjusting sample weights, the proposed loss effectively reduces both false positive and false negative rates.

## 2. Related Work

### 2.1. Research on Road Segmentation Algorithms

Road segmentation algorithms have evolved into three mainstream directions based on input modalities.

RGB-based methods have historically occupied a dominant position in this field. Encoder-decoder architectures, exemplified by Fully Convolutional Networks (FCN) [6] and U-Net [7], laid the foundational groundwork for semantic segmentation. Subsequently, Vision Transformers (ViT) and their segmentation variants—such as Seg-Former [8] and Mask2Former [9]—have significantly advanced performance by leveraging self-attention mechanisms to capture global context. Notably, the integration of Shuffle Attention [10] has markedly enhanced the representation of long-range dependencies, achieving substantial metric improvements on the CamVid dataset. Furthermore, to meet the stringent real-time requirements of autonomous driving while maintaining high accuracy, RoadNet-RT [11] employs a heterogeneous network architecture and efficient feature extraction modules, achieving an excellent trade-off between computational cost and performance. However, since these methods rely solely on color and texture cues, their performance ceiling—constrained by the absence of depth information—has become increasingly evident in complex environments.

Fusing LiDAR point clouds serves as a key solution to the robustness bottlenecks of vision-only schemes. These strategies are broadly classified into three levels. At the data level, methods like PV-RCNN [12] employ dense voxel encoding via 3D convolutions, though often at the cost of geometric fidelity. PLARD [13] advances this by adopting a progressive adaptation scheme, transforming LiDAR data into image-compatible features to mitigate inter-modal distribution shifts. Feature-level fusion remains the dominant paradigm: PointPainting [14] enriches point clouds with image-derived semantics, while FusionNet [15] aligns point features to the image plane. Moreover, Transformers have introduced powerful mechanisms for fusion, such as the adaptive cross-attention proposed by Gu et al. [16]. Decision-level fusion, which merges outputs via post-processing, offers stability but generally achieves suboptimal precision compared to feature-level integration.

Alternatively, RGB-D strategies [17] present a pragmatic compromise. The central challenge is the synergistic fusion of visual and geometric cues. SPNet [18] tackles sensor noise through a progressive network design. SNE-RoadSeg [19] leverages geometric priors by converting depth into surface normals, thereby explicitly encoding road gradients and flatness. USNet [20] accounts for data quality variance via an uncertainty-aware mechanism that adjusts weights according to the depth map’s SNR. However, a critical limitation persists: prevalent methods predominantly adopt standard RGB backbones. This approach neglects the specific need to capture high-frequency geometric boundaries—arising from sharp depth discontinuities—consequently limiting the model’s ability to resolve fine-grained details.

To systematically review the technological evolution of road segmentation, Table 1 provides a comparative summary of representative works across multiple dimensions, including methodologies, core concepts, feature extraction strategies, advantages, and limitations. This tabular analysis clearly delineates the prevailing technical trends and highlights the innovative positioning of the proposed work within the field.

### 2.2. On the Application of Wavelet Transform in Image Processing

As a robust tool for time-frequency analysis, the Wavelet Transform (WT) has garnered sustained attention in the field of image processing due to its ability to simultaneously provide frequency and spatial localization. Unlike the Fourier Transform, which yields only global frequency information, the WT decomposes signals at multiple scales using local basis functions, making it particularly adept at extracting local features such as edges and textures. In recent years, the integration of WT with deep learning has led to significant advancements across several dimensions:

1. Enhancing Feature Extraction: WT can explicitly extract high-frequency details and edge information. For instance, in low-light image enhancement, WaveletMamba [21] utilizes the Discrete Wavelet Transform (DWT) to decouple low-and high-frequency components, optimizing brightness enhancement and detail preservation in parallel. Similarly, MSCWNet [22] employs a dense wavelet network for multi-scale feature fusion, endowing the network with denoising capabilities while minimizing information loss.
2. Reducing Computational Complexity: When combined with methods [23,24] like Winograd, WT effectively lowers computational costs, offering distinct advantages for high-resolution image processing or deployment on resource-constrained platforms. Related studies utilizing grouped pixel processing strategies have significantly reduced asymptotic computational complexity.
3. Providing a Multi-scale Analytical Perspective: Wavelet decomposition inherently offers a multi-scale representation of images, which aligns perfectly with the hierarchical feature extraction philosophy of deep learning. WMANet [25], for example, decomposes images via DWT, processing high- and low-frequency components at different scales before reconstructing the enhanced image through inverse transformation.

In specific applications, Fan et al. [26] introduced WT into RGB-D semantic segmentation, decomposing depth maps into four sub-bands via DWT to explicitly separate global contours from detailed edges. Sun et al. [27] leveraged WT for multi-scale decomposition, designing differentiated processing paths for distinct frequency sub-bands. Wang et al. [28] proposed a wavelet-guided cross-modal attention mechanism, using high-frequency depth information to generate attention masks that guide the model to emphasize geometric edge regions. WaveFill [29] applied differential processing to frequency sub-bands in image inpainting, offering new insights for multi-modal tasks. More recently, WT has been integrated with the Mamba architecture; studies such as WMamba [30], IRSRMamba [31], and WaveletMamba [21] combine frequency domain separation with State Space Models (SSMs) to capture long-range dependencies, demonstrating promising potential.

In summary, leveraging its multi-resolution analysis capabilities, WT provides an effective technical approach for image processing. Current research focuses on optimizing frequency domain strategies, fusing with emerging architectures, and improving efficiency. Future exploration is likely to center on adaptively adjusting frequency band weights, further reducing complexity, and designing more efficient frequency domain processing modules.

### 2.3. Gating-Based Fusion Methods

Gating mechanisms, which dynamically modulate information flow via learnable units, have emerged as a pivotal strategy for achieving adaptive multi-modal fusion. By automatically calibrating the contribution of different modalities or features based on input content, these methods significantly enhance the robustness and flexibility of the fusion process.

Early research introduced gating concepts into multi-modal fusion; for instance, IGNFusion [32] utilizes a Sigmoid function to generate gating vectors for the weighted aggregation of multi-modal features. With the advancement of research, spatial-domain gated attention methods have evolved. Notably, SA-Gate [33] computes spatial attention maps to suppress interference from unreliable depth regions while bolstering the response of critical areas such as geometric edges, thereby effectively improving fusion quality in noisy environments. In the realm of channel-domain gating, the Squeeze-and-Excitation (SE) module proposed in SE-Net [34] has inspired a plethora of subsequent studies. Specifically, CFM [35] designed a channel-gated fusion module for RGB-D segmentation that generates channel attention weights by squeezing and exciting depth features to modulate RGB representations, significantly enhancing cross-modal feature complementarity.

Recently, cross-modal interactive gating has become a research hotspot. BiGRU [36] introduced a bidirectional gating mechanism where two modalities reciprocally generate gating signals for each other, effectively exploiting complementary information. Cutting-edge research has begun to synergize gating mechanisms with wavelet transforms. For example, WDM-UNet [37] pioneered the learning of independent gating weights for different wavelet sub-bands of depth maps to achieve frequency-domain adaptive fusion, significantly enhancing edge details while preserving structural information.

While gating-based fusion is evolving towards more fine-grained interactions and integration with emerging architectures, challenges regarding the interpretability of gating signals, computational efficiency, and stability under extreme scenarios remain to be fully addressed.

## 3. Methodology

In this section, we first provide an overview of the overall architecture of WG-FuseNet. Subsequently, we elaborate on its key components, specifically the Cross-Scale Enhanced Wavelet Transform Module and the Gated Attention-based Fusion Module. Finally, we present our improved loss function.

### 3.1. Overall Framework Design

**Figure 2:**
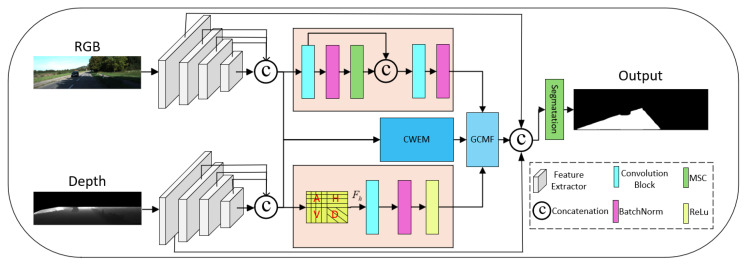
Architectural Overview of the Proposed WG-FuseNet Framework.

Figure 2 illustrates the overall architecture of the proposed WG-FuseNet for semantic segmentation. The network adopts a dual-encoder-single-decoder paradigm to process bi-modal inputs, namely RGB and depth images. Specifically, these inputs are fed into two parallel backbone networks with identical structures to extract multi-level hierarchical features.

Subsequently, a feature enhancement stage is employed, comprising three distinct branches designed to refine specific feature representations.

For RGB features, we construct a Multi-Scale Feature Pyramid Network. This branch performs channel adjustment and upsampling on deep, semantically rich features (e.g., x_r4, x_r3, x_r2) to align their scales with shallow, high-resolution features. Subsequently, features from different scales are concatenated to achieve comprehensive multi-scale fusion. Within the Multi-Scale Context (MSC) block, max pooling operations are employed at three distinct kernel sizes to capture multi-scale contextual information, formulated as:

(1) $$x^{\prime }=\left[{\mathrm{MP}}_{5\times 5}\left(x\right),\;{\mathrm{MP}}_{7\times 7}\left(x\right),\;{\mathrm{MP}}_{9\times 9}\left(x\right)\right]$$

where [,] indicates channel-wise concatenation.

For depth features, we introduce frequency-domain feature extraction. By leveraging the sensitivity of wavelet transforms to abrupt signal changes, this branch guides the model to focus specifically on sharp transitions corresponding to road edges.

Additionally, we propose the Cross-Modal Wavelet Enhancement Module. This module processes multi-scale RGB and depth features in the frequency domain, adaptively amplifying structural details along various orientations before reconstructing them via inverse wavelet transform.

Finally, to effectively integrate the aforementioned feature representations, we design a Gated Attention-based Fusion Module. This module is capable of adaptively learning the complementary relationships among RGB features, depth features, and the enhanced wavelet-transformed features, thereby enabling dynamic feature combination. For instance, the mechanism allows the model to rely more heavily on RGB information in texture-rich regions, while prioritizing depth cues in scenarios involving shadows, occlusions, or long-range distances. Ultimately, the fused features are fed into a segmentation head to be mapped into category prediction maps, which are then upsampled to the original input resolution (H, W) to generate the final road segmentation output.

### 3.2. Cross-Scale Wavelet Enhancement Module

**Figure 3:**
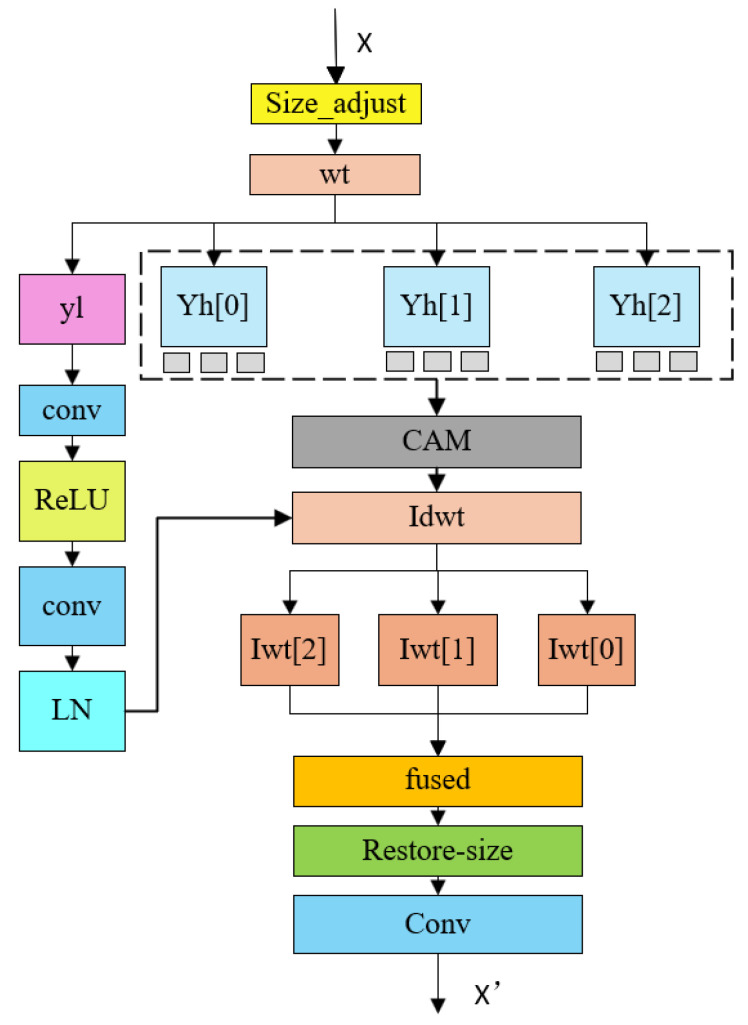
Cross-scale Enhanced Wavelet Module (CWEM).

To tackle the dual challenges of insufficient multi-modal feature fusion and the loss of fine-grained details in RGB-D road segmentation, we propose the Multi-scale Wavelet Enhancement Module (MWEM). As depicted in Figure 3, the core strategy of this module is to leverage wavelet transforms to decompose features into distinct frequency sub-bands. We then perform targeted enhancement on these sub-bands, tailored to the semantic characteristics of road scenes. This approach effectively amplifies edge and texture details—which are crucial for segmentation accuracy—while simultaneously preserving global structural information.

Specifically, given an input feature map, we first perform spatial alignment by padding the input to the nearest power-of-two dimensions to satisfy the requirements of the wavelet transform. This padding operation is defined as follows:

(2) $$X^{\prime }=P_{r}\left(X,(0,\Delta W,0,\Delta H)\right)$$

where $\Delta W=2^{\left\lceil \log_{2}\;W\right\rceil }-W$, $\Delta H=2^{\left\lceil \log_{2}H\right\rceil }-H$, $P_{r}$ indicates reflection padding.

Subsequently, a 3-level discrete wavelet transform (DWT) is applied to X’, decomposing it into one low-frequency component YL (the approximation coefficients at level 3) and three high-frequency component lists YH[i] (each containing detail coefficients in three orientations: horizontal LH, vertical HL, and diagonal HH). We employ the db4 wavelet basis to effectively capture smooth transitions while maintaining computational efficiency and decoupling capability.

The low-frequency component encapsulates the global structural and semantic information of the scene. We enhance this component using a lightweight encoder-decoder network—comprising two 3 × 3 convolutional layers and a bilinear upsampling layer—designed to refine and strengthen the low-frequency contextual information relevant to road structures.

Conversely, the high-frequency components contain rich edge and texture details essential for precise segmentation. To exploit these features, we design a frequency-band attention mechanism that applies Channel-Spatial Attention Modules to the three directional high-frequency sub-bands. This enables the network to adaptively focus on critical edges and textures while suppressing noise or irrelevant artifacts. The enhancement process proceeds as follows:

(3) $$x^{\prime }=\mathrm{sa}\left(\left[x_{{\mathrm{ca}}_{\mathrm{mean}}},x_{{\mathrm{ca}}_{\max}}\right]\right)⊙x_{\mathrm{ca}}$$

where x_ca=(ca(x)⊙x), and $\left[,\right]$ denotes channel-wise concatenation.

Next, we proceed to feature map reconstruction. Commencing from the coarsest scale, the enhanced low-frequency and high-frequency components are progressively synthesized via the Inverse Discrete Wavelet Transform (IDWT) to restore feature maps to their original resolution. This iterative process yields reconstructed representations at three distinct scales.

To effectively leverage these multi-scale features, a cross-scale fusion strategy is applied. We integrate these reconstructed maps through upsampling layers and convolutional fusion blocks. This mechanism achieves the complementary integration of multi-scale information. The specific implementation is formulated as follows:

(4) $$\mathrm{Fused}={\mathrm{FB}}^{2}\left({\mathrm{FB}}^{1}\left({\mathrm{FB}}^{0}\left(F^{2}⊕U^{0}\right)⊕U^{1}\right)⊕U^{2}\right)$$

where $F^{2}$ denotes the reconstructed feature map at the finest scale, and the formulas for ${\mathrm{FB}}^{i}\left(F⊕U^{i}\right)$ and $U^{i}$ are defined as follows:

(5) $${\mathrm{FB}}^{i}\left(F⊕U^{i}\right)=\mathrm{Relu}\left(\mathrm{BN}\left({\mathrm{conv}}_{3*3}\left(\left[F,U^{i}\right]\right)\right)\right)$$

(6) $$U^{i}=\mathrm{Upsample}\left({\mathrm{lwt}}^{i},T\right)$$

where *T* denotes the target size at the finest scale, and [,] represents channel-wise concatenation. Finally, size restoration and output are performed: the fused feature map is cropped back to its original dimensions, followed by a convolutional layer for final transformation and dimensionality reduction, ultimately outputting the enhanced features.

### 3.3. Gated Cross-Modal Fusion Module

**Figure 4:**
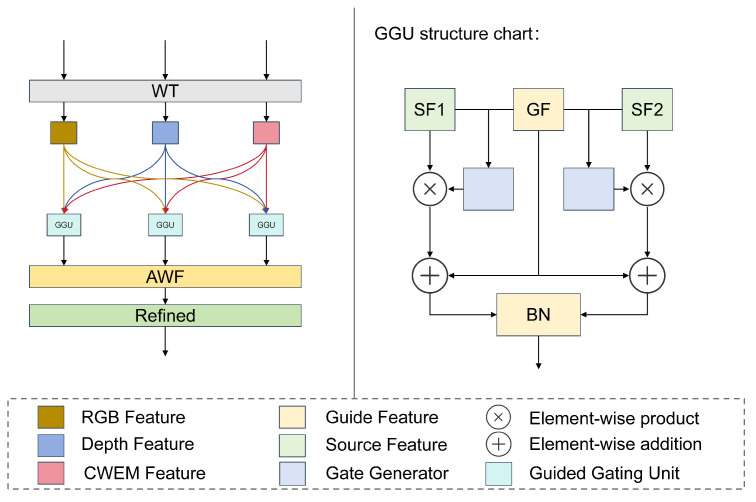
Gated Cross-Modal Fusion Module (GCMF).

To effectively integrate multi-source heterogeneous information and accentuate critical road features, we propose a Gated Cross-Modal Fusion (GCMF) module based on gated attention. As illustrated in Figure 4, this module facilitates adaptive fusion of multi-modal features through a hierarchical processing pipeline. Initially, we independently transform the three input feature representations, mapping them into a unified feature space. This alignment ensures that all modalities interact within a consistent semantic domain for subsequent operations. Specifically, each modality is processed by a dedicated feature transformation function:

(7) $$F_{i}^{\mathrm{trans}}=\mathrm{Relu}\left(\mathrm{BN}\left({\mathrm{conv}}_{3*3}\left(F_{i}\right)\right)\right),\;i\in \left\{\mathrm{RGB},\mathrm{Depth},\mathrm{Wavelet}\right\}$$

Next, we design a bidirectional gating unit to facilitate fine-grained information interaction between modalities. This mechanism empowers each modality to selectively incorporate complementary information from others while preserving the integrity of its own features. Given any pair of transformed modal features $F_{i}^{\mathrm{trans}}$ and $F_{j}^{\mathrm{trans}}$, the interaction process can be formulated as follows:

(8) $$G_{i\to j}=\sigma \left({\mathrm{conv}}_{1*1}\left(\left[F_{i}^{\mathrm{trans}},F_{j}^{\mathrm{trans}}\right]\right)\right)$$

(9) $$F_{i}^{\mathrm{enhanced}}=F_{i}^{\mathrm{trans}}+G_{i\to j}⊙F_{j}^{\mathrm{trans}}$$

where σ denotes the Sigmoid function, producing attention weights in the range [0,1], ⊙ represents element-wise multiplication, and [,] indicates channel-wise concatenation, *i*, *j* represents any two modes, and $G_{i\to j}$ represents the gain of *i* mode to *j* mode.

We construct six gated generator to facilitate full bidirectional communication among all modal pairs. This design guarantees that each modality maximizes the assimilation of complementary cues from the others.

**Figure 5:**
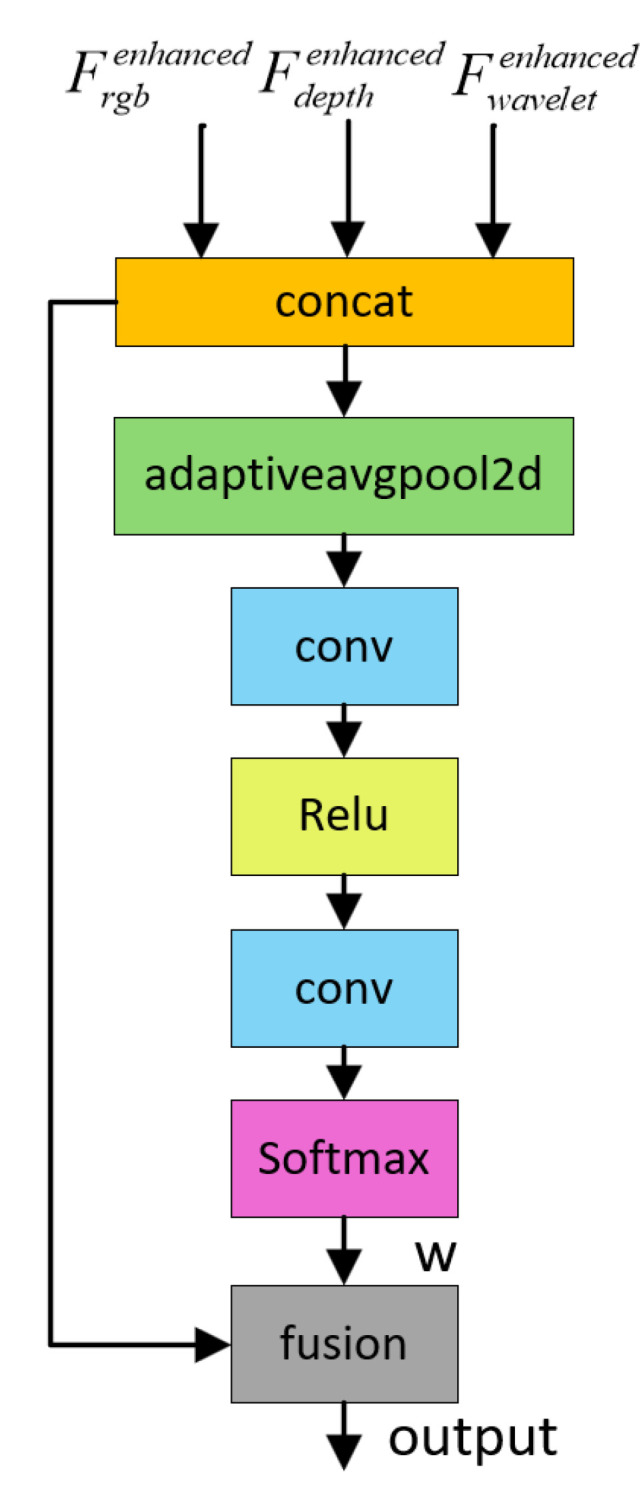
Adaptive Weight Generation Module (AWF).

Subsequently, to synthesize these features, we deploy an Adaptive Weighting Fusion (AWF) mechanism (Figure 5) that dynamically recalibrates the contribution of each modality based on its global importance. Within this module, Global Average Pooling is employed to distill global context, which is then processed by a two-layer Multi-Layer Perceptron to learn non-linear inter-modal relationships. The derived weights guide the weighted aggregation of the features:

(10) $$\mathrm{output}=\sum w_{\tilde{i}}*F_{\tilde{i}}^{\mathrm{enhanced}},\;i\in \left\{\mathrm{RGB},\mathrm{Depth},\mathrm{Wavelet}\right\}$$

where $w_{i}$ represents the weight of the *i* mode.

Finally, feature refinement is applied to the aggregated representation. A convolutional layer is employed to perform dimensionality reduction and non-linear transformation, compressing the channel dimension from in_channels×3 to decode_channels while simultaneously enhancing feature representation capability. The resulting road-aware feature map is subsequently utilized for the downstream segmentation task.

### 3.4. Loss Function

We observe that most existing road segmentation algorithms can adequately identify the main drivable regions. However, these methods frequently produce false positive predictions in non-drivable areas that share high visual similarity with roads, such as sidewalks and opposite lanes. Notably, this phenomenon often overshadows the issue of incomplete detection (false negatives) in actual road regions. To address this, we design a loss function that incorporates a specific penalty mechanism to suppress such erroneous detections.

The Focal Loss function, widely adopted in segmentation tasks, is defined as:

(11) $$L_{\mathrm{FL}}=-\frac{1}{N}\sum_{i=1}^{N}\left[\alpha {\left(1-p_{i}\right)}^{\gamma }y_{i}\log\left(p_{i}\right)+\left(1-\alpha \right)p_{i}^{\gamma }\left(1-y_{i}\right)\log\left(1-p_{i}\right)\right]$$

where $p_{i}$ is the predicted probability, α is a balancing parameter set to 0.25 in our method, γ is the focusing parameter set to 2, and ${\left(1-p_{i}\right)}^{\gamma }$ serves as the modulating factor that reduces the weight of easily classified examples.

To mitigate false detections in non-drivable regions that resemble roads (e.g., sidewalks and opposing lanes), we incorporate a penalty mechanism into the loss function. By introducing a specific false detection penalty term, we explicitly suppress incorrect learning patterns associated with these misclassifications. The penalty term is formulated as follows:

(12) $$L_{\mathrm{FP}}=\lambda *R_{\mathrm{fp}}=\lambda *\frac{\sum_{i=1}^{N}I\left(p_{i}>0.5\wedge y_{i}=0\right)}{\sum_{i=1}^{N}I\left({\tilde{y}}_{i}\neq \mathrm{ignore}\right)}$$

where λ denotes the penalty coefficient, $R_{fp}$ represents the false positive rate, and *I* is an indicator function used to count the road regions that satisfy specific conditions.

Finally, we integrate the focal loss with the penalty term to form the complete loss function as follows:

(13) $$L_{total}=L_{FL}+L_{FP}$$

## 4. Result

In this section, we first introduce the datasets and evaluation metrics in Section 4.1 and Section 4.2. Implementation details regarding network training are provided in Section 4.3. Subsequently, Section 4.4 reports the quantitative and qualitative results of our method. Finally, ablation studies analyzing each component are presented in Section 4.5.

### 4.1. Dataset

KITTI_Road Dataset [38]: This study employs the KITTI_Road dataset for model training and performance evaluation. As one of the most widely adopted benchmarks for road segmentation, this dataset encompasses images with rich scene variations, diverse lighting conditions, and complex road structures, thereby rigorously testing the robustness and generalization capability of algorithms in real-world scenarios. The dataset comprises 289 training samples and 290 test samples, covering three distinct road scene categories: Urban Marked (UM), Urban Multiple Marked (UMM), and Urban Unmarked (UU). For our experiments, we partition the official training samples into training and validation subsets at a 70:30 ratio, using the validation set to monitor performance.

ORFD Dataset [39]: Additionally, we utilize the ORFD dataset, which contains 30 image sequences capturing off-road environments across various seasons and weather conditions. The dataset consists of 12,198 LiDAR-RGB image pairs with corresponding pixel-level road labels. These are divided into training, validation, and testing sets following a ratio of 70:10:20.

### 4.2. Evaluation Metrics

We adhere to the standard evaluation metrics established for the KITTI benchmark [38]. The metrics include Maximum F-score (MaxF), Precision (PRE), Recall (REC), False Positive Rate (FPR), and False Negative Rate (FNR). Additionally, we incorporate Intersection over Union (IoU) and Accuracy (Acc) as supplementary evaluation criteria. The computational formulas for these metrics are defined as follows:

Maximum F-score:

(14) $$\mathrm{MaxF}=\frac{2*(\mathrm{Precision}*\mathrm{Recall})}{\mathrm{Precision}+\mathrm{Recall}}$$

Precision:

(15) $$\mathrm{Precision}=\frac{t_{p}}{t_{p}+f_{p}}$$

Recall:

(16) $$\mathrm{Recall}=\frac{t_{p}}{t_{p}+f_{n}}$$

Iou:

(17) $$\mathrm{Iou}=\frac{t_{p}}{t_{p}+f_{p}+f_{n}}$$

Accuracy:

(18) $$\mathrm{Accuracy}=\frac{t_{p}+t_{n}}{t_{p}+f_{p}+t_{n}+f_{n}}$$

### 4.3. Implementation Details

Our experiments were conducted on an Ubuntu 22.04 operating system, powered by an Intel i7-9700K CPU (Intel, Santa Clara, CA, USA) and an NVIDIA GeForce RTX 3090 GPU (Nvidia, Santa Clara, CA, USA). The software environment was established using Python 3.10 and the PyTorch 2.3.1 deep learning framework, with CUDA 11.8 enabled for GPU acceleration.

For the network architecture, we employed a ResNet-18 model pre-trained on ImageNet as the backbone feature extractor. Input images were uniformly resized to 1248 × 384 pixels, and the batch size was set to 2. We utilized the AdamW optimizer with an initial learning rate of 1 × 10^−3^. A cosine annealing strategy was adopted to gradually decay the learning rate to a minimum of 8 × 10^−6^ over a total of 100 training epochs. The model was optimized using the proposed improved loss function.

To further bolster the model’s generalization capability and robustness in complex scenarios, a multi-level data augmentation strategy was implemented during the training phase. Specifically, for spatial transformation, we applied random horizontal flipping (probability of 0.5), random cropping (scale range 0.8–1.2), random rotation (angle range ± 5), and random affine transformations. Regarding photometric augmentation, we employed random brightness adjustment (20%), Gaussian noise injection (σ≤0.02), and Gaussian blurring (kernel size 3–7). All augmentation operations were executed online in real-time, ensuring sample diversity across epochs and effectively enhancing the model’s adaptability to real-world disturbances such as illumination changes, viewpoint shifts, and sensor noise.

### 4.4. Comparative Experiments

To comprehensively evaluate the effectiveness of our proposed method, we selected state-of-the-art road segmentation networks across different sensor modalities as baselines. These comparisons include algorithms based on pure RGB images, RGB-LiDAR fusion, and RGB-Depth fusion.

1. RoadNet-RT [11] proposes a lightweight, high-throughput convolutional neural network architecture optimized for hardware accelerators through techniques like depthwise separable convolutions and non-uniform kernels to achieve accurate, re-al-time processing speeds.
2. LFD_RoadSeg [1] operates on the premise that low-level features are more feasible for road representation than high-level semantics. It designs a spatial detail branch to extract low-level representations and introduces a contextual semantic branch to suppress textureless regions often mistaken for roads. A selective fusion module then computes pixel-level attention to filter non-road responses.
3. LidCamNet [40] projects unstructured point cloud information onto the image plane, followed by upsampling to obtain dense 2D image sets encoding spatial information. Multiple fully convolutional neural networks are then employed for road segmentation.
4. PLARD [13] transforms LiDAR data into visual data space through height difference transformation to achieve data space adaptation, using a cascaded fusion structure to adaptively align LiDAR features with visual features for feature space adaptation. This enables progressive LiDAR-adaptive assisted road detection by gradually adapting LiDAR information into vision-based road detection.
5. SNE-RoadSeg [19] introduces a novel module that efficiently infers surface normal information with high accuracy from dense depth/disparity images. It then employs a data-fusion CNN architecture to extract and fuse features from RGB images and the inferred surface normal information for precise free-space detection.
6. Usnet [20], based on evidence theory, eliminates the essential cross-modal feature fusion operations previously required in RGB-D methods. It uses two lightweight sub-networks to learn road representations from RGB and depth inputs, and designs a multi-scale evidence collection module to gather evidence at multiple scales for each modality, providing sufficient evidence for pixel-level classification. Finally, an Uncertainty-Aware Fusion (UAF) module perceives each modality’s uncertainty to guide the fusion of the two sub-networks.

For comparative algorithms with publicly available results on the relevant datasets, we directly cite the reported metrics. For those without published results, we reproduced the algorithms using their official implementations. Through rigorous comparison with these representative methods on the KITTI road benchmark, we demonstrate the superiority of our approach in terms of both accuracy and real-time performance.

Table 2 presents the quantitative experimental results of our method on the KITTI_Road dataset. As detailed in the table, our approach achieves a MaxF of 97.31%, ranking highest among all comparative methods, which underscores its overall superiority. Furthermore, our method excels in both Precision and Recall metrics, indicating a well-balanced capability in mitigating both false negatives (missed detections) and false positives (false detections). Crucially, regarding processing speed—a critical performance metric in autonomous driving scenarios—our method also demonstrates outstanding performance with a per-frame processing time of only 22.94 ms, significantly outperforming most comparative approaches and exhibiting promising real-time processing capability and practical deployment potential.

**Figure 6:**
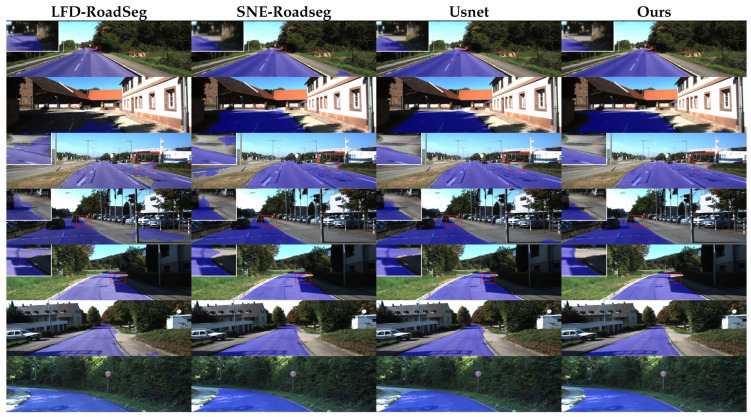
Qualitative results on the KITTI-Road test set. The blue regions indicate predicted road areas. Red dashed circles highlight incorrect segmentation regions. Some inconspicuous areas were enlarged.

Figure 6 presents the qualitative analysis results of various road segmentation algorithms. Visual inspection reveals that while all methods achieve satisfactory performance in conventional scenarios characterized by favorable lighting and distinct road structures, most comparative methods exhibit noticeable false negatives and false positives in challenging areas, such as road edges, sidewalk boundaries, and shadowed regions. Of particular concern is that several baseline algorithms misclassify non-drivable curbs as drivable road surfaces—a critical perception error that could pose severe safety hazards in real-world autonomous driving applications. In contrast, our proposed method maintains superior stability and accuracy across diverse complex scenarios. It effectively suppresses boundary ambiguities and shadow interference, thereby demonstrating enhanced robustness and practical utility.

Table 3 details the experimental results of various algorithms across distinct road scenarios. The proposed method demonstrates consistently superior and well-balanced performance across all three sub-tasks of the KITTI Road benchmark. Specifically, in Urban Multiple Marked (UMM) and Urban Unmarked (UU) road scenarios, our approach significantly outperforms comparative models. This strongly validates that the learned road features possess excellent generalization capabilities, enabling the effective handling of complex and unstructured environments. While in the relatively structured Urban Marked (UM) road scenario, our method performs slightly lower than USnet, its demonstrated advantages in both UMM and UU scenarios indicate better robustness and generalization capability. In summary, compared to existing state-of-the-art methods, our solution maintains high efficiency while achieving improved accuracy, particularly establishing a significant advantage in cross-scenario robustness.

Table 4 presents the quantitative results on the ORFD dataset, assessing the model’s generalization capability in unstructured off-road environments. Beyond standard RGB benchmarks, we explicitly investigated the impact of the HSV color space. The rationale behind this design choice lies in the inherent characteristics of off-road scenes, which are frequently plagued by erratic illumination, strong shadows, and reduced texture contrast between the road and the background. Unlike RGB, where chromaticity and intensity are entangled, HSV separates luma (brightness) from chroma. This separation theoretically provides an illumination-invariant representation, allowing the model to distinguish road materials based on intrinsic color properties rather than susceptible intensity values.

As evidenced in Table 4, the integration of HSV yields performance gains for methods capable of effective feature fusion. SNE-RoadSeg observes a moderate improvement of 0.37% in MaxF. Our proposed method, leveraging its advanced Gated Cross-Modal Fusion (GCMF) module, successfully exploits this complementary spectral information, achieving a further performance boost of 0.63% and securing a state-of-the-art MaxF of 96.49%. Interestingly, OFF-Net exhibits a marginal performance decline (−0.41%) when transitioned to the HSV domain. This suggests that its architecture may be predominantly reliant on RGB-specific texture patterns. Overall, our method significantly outperforms all comparators, demonstrating superior adaptability.

**Figure 7:**
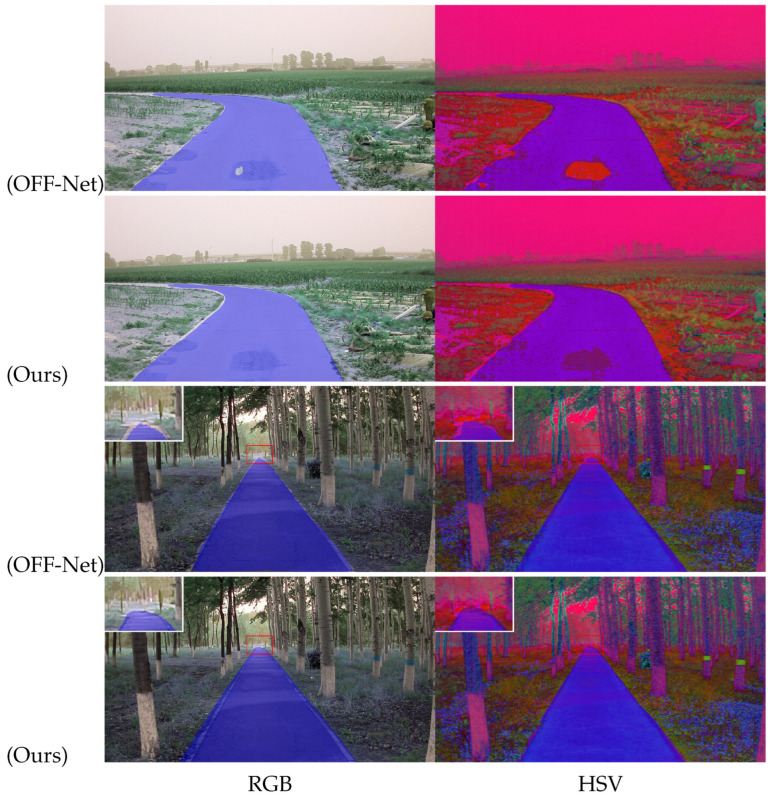
Qualitative comparison between RGB and HSV color spaces on the ORFD dataset. Results using RGB and HSV are shown in the left and right columns, respectively.

Qualitatively, Figure 7 visualizes the segmentation challenges inherent in the ORFD dataset. In the first scenario, the baseline OFF-Net erroneously classifies the dark-pigmented road surface as a non-road region. Similarly, in the second scenario, it suffers from incomplete detection regarding the distant road terminus. In contrast, our proposed method successfully circumvents these pitfalls. By effectively fusing spatial contextual information, our model precisely delineates road boundaries even in visually ambiguous regions, compellingly validating its reliability in complex, unstructured environments.

### 4.5. Ablation Studies

To verify the effectiveness of each proposed component, we conducted systematic ablation studies on the KITTI_Road dataset, with results summarized in Table 5. First, compared to the uni-modal baselines (RGB and Depth), a simple dual-modal fusion strategy boosts the F-score to 95.28%. This confirms the necessity of fusing RGB and depth information, although there remains substantial room for performance improvement.

Subsequently, we incrementally introduced the core modules. By effectively expanding the receptive field, the Multi-Scale Context (MSC) block boosts the Precision to 95.86%. The integration of the Cross-Scale Wavelet Enhancement Module (CWEM) significantly elevates the F-score to 96.83%. Notably, the Recall metric improves from 95.76% to 97.31%, indicating that this module—through multi-scale frequency analysis—effectively enhances the perception of road boundaries and hard samples, thereby significantly reducing false negatives. Conversely, the Gated Cross-Modal Fusion (GCMF) module exhibits a distinct optimization tendency, lifting the F-score to 96.37%. This module increases Precision from 94.81% to 95.89%, demonstrating its ability to intelligently integrate multi-modal information via an adaptive gating mechanism. It effectively suppresses false positives while maintaining high recall, thus enhancing the reliability of the segmentation results.

Furthermore, the validation of our improved loss function confirms its efficacy in suppressing erroneous detections and improving precision. Ultimately, our complete model, which integrates all modules, achieves optimal performance. This demonstrates not only the individual effectiveness of the proposed modules but also the strong complementarity among them.

**Figure 8:**
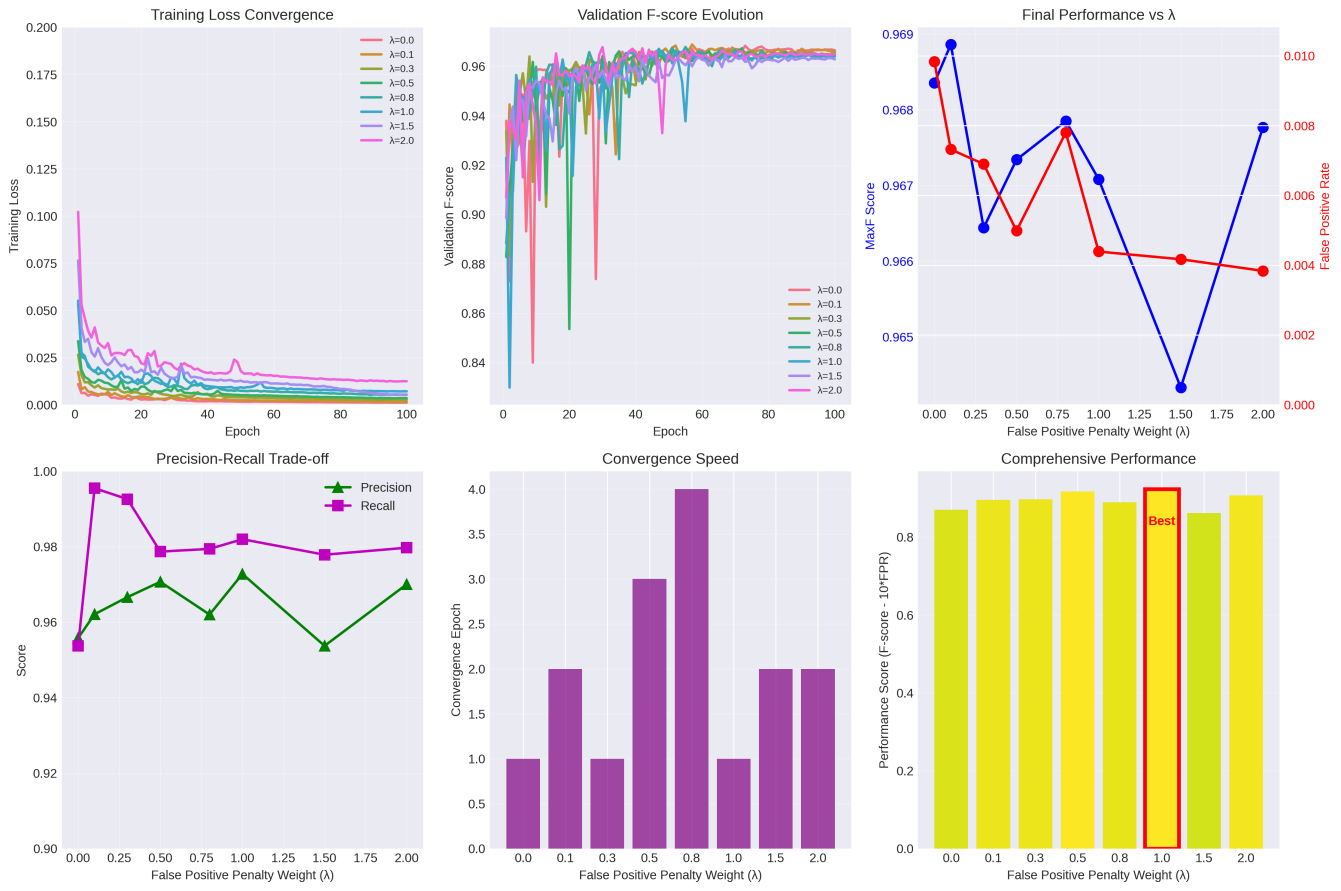
Impact of the False Detection Penalty Weight.The sub-figures display: training loss convergence, validation F-scores, final performance metrics (MaxF and FPR), the precision-recall trade-off, convergence speed, and comprehensive performance indicators.

To investigate the sensitivity of the false positive penalty coefficient λ in our loss function, we conducted extensive experiments on the KITTI-Road dataset by varying λ from 0 to 2.0. The models were trained for 100 epochs, with detailed visualizations provided in Figure 8.

The impact of λ on the optimization process is multifaceted. As shown in the “Training Loss Convergence” (top-left) and “Convergence Speed” (bottom-middle) plots, the penalty term alters the convergence behavior. While moderate penalties (e.g., λ=0.8) extend convergence time—suggesting the model actively navigates the trade-off—excessively high penalties (e.g., λ=2.0) result in higher training loss and instability, indicating disruption in the optimization landscape.

Regarding detection performance, the “Final Performance vs. λ” (top-right) plot reveals a critical trade-off. The False Positive Rate (FPR, red line) consistently decreases as λ increases, confirming that the penalty term effectively suppresses misclassifications in non-drivable regions. However, the MaxF score (blue line) fluctuates, with a sharp decline at λ=1.5, suggesting that overly aggressive penalties may hinder road feature learning. This observation is corroborated by the “Precision-Recall Trade-off” (bottom-left) plot, where λ=1.0 achieves the highest precision before performance degradation.

To quantitatively determine the optimal hyperparameter, we employed a comprehensive performance metric ($F_{\mathrm{score}}-10\times \mathrm{FPR}$). As highlighted in the bottom-right bar chart, the model attains the highest comprehensive score at λ=1.0 (marked as “Best”). This configuration optimally balances segmentation accuracy (MaxF) and false alarm suppression (FPR). Consequently, λ=1.0 is adopted as the default coefficient for our proposed method.

Then, we systematically investigate the impact of different wavelet bases and decomposition levels on model performance, with results tabulated in Table 6.

First, regarding the wavelet basis, the Haar wavelet acts as a discontinuous step function. While effective at detecting abrupt signal changes, it is prone to introducing “blocking artifacts” during feature reconstruction. In road scenes, critical features such as curbs and lane markings are typically continuous, smooth curves. The inherent discontinuity of the Haar wavelet fails to preserve this geometric smoothness, resulting in jagged boundaries in the segmentation output. In contrast, the DB4 wavelet possesses higher vanishing moments and superior regularity. This allows it to better approximate the polynomial-like variations found in road surfaces and textures. This characteristic enables the CWEM to capture subtle gradations in road texture while simultaneously maintaining the integrity of continuous geometric boundaries, thereby yielding superior Precision and Recall.

Second, regarding the decomposition level (J), at lower levels (J = 1, 2), the receptive field is insufficient to fully differentiate high-frequency noise (e.g., gravel texture, camera noise) from structural edges. Conversely, at higher levels (J = 4), the resolution of the low-frequency component drops to 1/16 of the original size. Since semantic segmentation necessitates pixel-level precision, such excessive downsampling causes an irreversible loss of fine spatial information, leading to the vanishing of small obstacles or distant road ends—as evidenced by the decline in Recall. Level J = 3 strikes the optimal balance: it ensures that low-frequency components capture a coherent global semantic structure, while high-frequency components effectively extract structural boundaries.

Finally, the ablation results in Table 6 clarify that the effectiveness of CWEM stems from its ability to process road features in the frequency domain. Low-frequency components contribute to shadow robustness. Road surfaces typically exhibit consistent low-frequency energy profiles. Since shadows primarily affect pixel intensity without altering physical texture, explicitly enhancing these components helps the network learn illumination-invariant representations, effectively mitigating the shadow misclassification issues highlighted. High-frequency components serve as boundary refiners. The channel-spatial attention mechanism within CWEM functions as a frequency filter, amplifying these structural edge responses while attenuating irregular high-frequency noise. This mechanism directly contributes to the improved road edge integrity observed in our experiments.

To thoroughly validate the design effectiveness of the Gated Cross-modal Fusion Module (GCMF), we conducted internal ablation studies, with results summarized in Table 7. First, removing the interactive gating mechanism (denoted as Fusion_RG) leads to significant performance degradation, confirming that our proposed bidirectional interaction mechanism is crucial for achieving fine-grained, spatially adaptive fusion. Second, eliminating the adaptive weighting component (denoted as Fusion_RA) also causes noticeable performance deterioration, demonstrating that dynamically balancing the global contributions of each modality is essential for model robustness.

**Figure 9:**
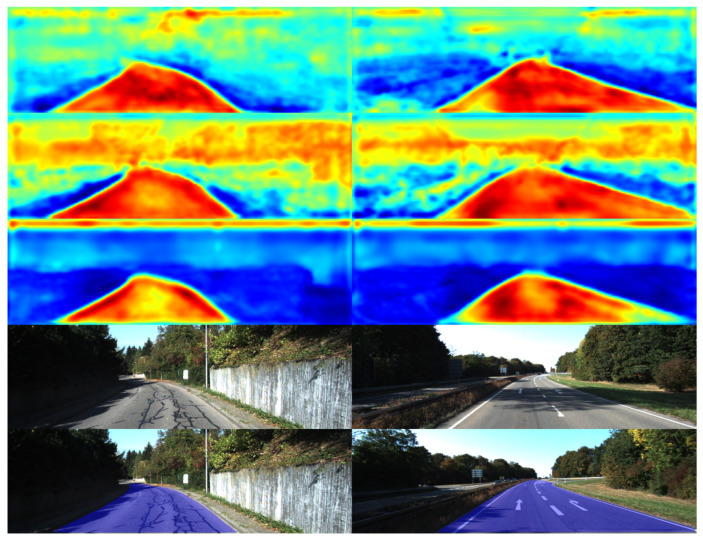
Visualization of attention weight distributions in the GCMF module. The top three rows display the refined RGB, Depth, and CWEM features, respectively, which have incorporated complementary information from other modalities. The fourth row presents the original RGB inputs, while the fifth row illustrates the final segmentation results generated by our algorithm.

To visually elucidate the internal working mechanism of the proposed Gated Cross-Modal Fusion (GCMF) module and empirically validate the complementarity between heterogeneous modalities, we visualized the gating weights (attention maps) learned during the inference phase. As illustrated in Figure 9, the heatmaps depict the spatial distribution of attention weights generated by the gating mechanism, where red regions signify high importance (feature enhancement) and blue regions indicate suppression. These visualizations reveal three distinct interaction patterns that align perfectly with our design philosophy:

First, regarding the depth features enriched with cross-modal information (Figure 9, Row 1), the attention heatmap exhibits pronounced geometric boundary sharpening. Although raw depth data often suffers from blurred edges due to sensor noise or resolution constraints, the fused features demonstrate extremely high activation intensity at road boundaries. This indicates that the GCMF module successfully guides the depth branch to assimilate high-frequency details from RGB textures, thereby rectifying blurred geometric contours and rendering the structural boundaries of the road distinct and precise.

Second, for the refined RGB features (Figure 9, Row 2), the attention map displays high regional semantic consistency. This is characterized by extensive, flat, and continuous red activation regions spanning the entire drivable surface. This distribution pattern implies that the network leverages the geometric planar consistency of depth information as a prior to effectively “fill in” texture discontinuities in RGB images caused by shadows, stains, or uneven illumination.

Furthermore, the Cross-Scale Wavelet Enhancement (CWEM) features (Figure 9, Row 3) demonstrate the dual advantages of frequency-domain synergy and background suppression. As observed, CWEM features not only preserve the main road structure from low-frequency components (manifested as coherent regional activation) but also precisely capture fine edge information from high-frequency components. Crucially, the vast deep blue areas indicate that the module exerts robust suppression on non-road regions (e.g., sky, grass, vertical obstacles). This confirms that CWEM acts as an efficient frequency-domain filter, significantly boosting the signal-to-noise ratio and enabling the network to focus on the most discriminative road features.

In summary, the visualization results compellingly prove that the GCMF module is not merely a simple linear superposition of features, but rather establishes an interpretable and adaptive synergistic mechanism. This complementary interaction is the key factor enabling WG-FuseNet to maintain high robustness and accuracy in complex, unstructured environments.

## 5. Conclusions

The multi-modal road segmentation model proposed in this paper effectively combines the rich texture of RGB images with the key geometric features of depth information through the wavelet transform enhancement module and the gated cross-modal fusion module, while significantly suppressing false detections with a specially designed loss function. Experiments on the KITTI_Road dataset validate the effectiveness of the proposed method—achieving excellent results of 97.31% and 99.04% on the two key metrics, MaxF and Acc, respectively—particularly demonstrating outstanding segmentation accuracy in complex scenes and edge regions, thereby confirming the effectiveness of the approach presented in this paper. Future research di-rections will explore self-supervised or weakly supervised learning strategies to reduce the model’s reliance on large-scale fine-grained annotated data, while further investigating robust fusion mechanisms for multi-modal data under extreme weather or poor lighting conditions.

## Figures and Tables

**Table 1:** Comparative Analysis of Road Segmentation Methodologies.

Input Modalities	Method	Core Concept	Feature Extraction	Strengths	Limitations
RGB-only Methods	RoadNet-RT	Lightweight CNN + DSC	Multi-scale convolutional features	Real-time optimization; hardware design	Lack of depth cues; poor performance in shadows
	LFD_RoadSeg	Low-level representation + selective fusion	Low-level textural feature extraction	Focus on importance of low-level features	Prone to misclassification in texture-similar regions
LiDAR-Fusion Methods	LidCamNet	Point cloud projection + FCN fusion	Sparse point cloud densification	Pioneering early-stage fusion framework	Inadequate handling of point cloud sparsity
	PLARD	Progressive LiDAR adaptation	Dual adaptation of data and features	Robust dual-adaptation fusion mechanism	High computational overhead
RGB-D Methods	SNE-RoadSeg	Surface Normal Estimation + CNN	Depth-based normal generation	Incorporation of geometric surface normal cues	Accuracy limited by normal estimation accuracy
	Usnet	Uncertainty-aware fusion	Multi-scale evidence collection	Avoidance of explicit feature fusion	Prone to sensor noise; insufficient depth feature learning

**Table 2:** Comparative Results of State-of-the-Art Algorithms on KITTI Road Benchmark. The upward arrow (↑) denotes that a higher value is better, whereas a downward arrow (↓) signifies that a lower value indicates superior performance. Bold values indicate the best results in the corresponding metric.

Method	Input	MaxF↑	Pre↑	Recall↑	Fpr↓	Fnr↓	Iou↑	Acc↑	Runtimes (s)↓
RoadNet-RT [11]	RGB	92.55	92.94	92.16	3.86	7.84	89.63	93.35	0.09
LFD RoadSeg [1]	RGB	95.21	95.35	95.08	2.56	4.92	93.21	95.22	**0.01**
LidCamNet [40]	RGB + Lidar	96.03	96.23	95.83	2.07	4.17	92.89	97.45	0.15
PLARD [13]	RGB + Lidar	97.03	97.19	96.88	1.54	3.12	93.10	98.06	1.50
SNE-RoadSeg [19]	RGB + Depth	96.75	96.90	96.61	1.70	3.39	93.11	98.72	0.10
Usnet [20]	RGB + Depth	96.89	96.51	97.27	1.94	2.73	**94.82**	**99.06**	0.02
Ours	RGB + Depth	**97.31**	**97.05**	**97.57**	**0.64**	**2.43**	94.75	99.04	0.02

**Table 3:** Performance Comparison of State-of-the-Art Algorithms Across Different Tasks on the KITTI Benchmark. Bold values indicate the best results in the corresponding metric.

	UM		UMM		UU	
Algorithms	MaxF	AP	MaxF	AP	MaxF	AP
RoadNet-RT [11]	93.20	88.82	92.85	90.58	91.60	88.94
LFD_RoadSeg [9]	95.73	91.89	95.45	93.67	94.43	92.17
LidCamNet [40]	96.58	92.95	96.28	94.72	95.23	93.01
PLARD [13]	97.38	93.86	97.05	95.65	96.06	94.28
SNE_RoadSeg [19]	96.95	93.12	96.58	94.84	95.60	93.55
Usnet [20]	**97.58**	**94.06**	97.29	95.81	96.21	94.73
Ours	97.52	93.68	**97.56**	**96.10**	**96.54**	**95.05**

**Table 4:** Quantitative results on the ORFD dataset. Bold values indicate the best results in the corresponding metric.

	MaxF (%)	Pre (%)	Recall (%)	Fpr (%)	Fnr (%)	Acc (%)
SNE-RoadSeg [19]	93.26	90.59	94.00	2.43	3.70	94.01
SNE-RoadSeg+HSV	93.67 (+0.37)	91.13	94.37	2.12	3.42	94.35
OFF-Net [39]	94.64	93.80	95.24	1.97	3.28	95.88
OFF-Net+HSV	94.23 (−0.41)	94.16	93.58	2.98	3.17	95.62
Ours	95.86	94.25	**98.06**	1.29	**1.04**	97.90
Ours+HSV	**96.49 (+0.63)**	**95.05**	97.97	**1.11**	2.03	**98.73**

**Table 5:** Ablation Study on Component Contributions of WG-FuseNet.

Module	MaxF (%)	Pre (%)	Recall (%)	Acc (%)
RGB	93.50	93.10	93.90	97.80
Depth	92.20	91.80	92.60	97.50
RGB-D	95.28	94.81	95.76	98.26
RGB-D+MSC	95.86	95.68	95.64	98.72
RGB-D+CWE	96.83	96.35	97.31	98.88
RGB-D+GCMF	96.37	95.89	96.85	98.74
RGB-D+Mpt-loss	96.29	96.76	95.32	98.68
Ours	97.25	96.89	97.86	99.01

**Table 6:** Ablation Study on Wavelet Basis Functions and Decomposition Bases.

Wavelet Basis Functions	Decomposition Levels	MaxF (%)	Pre (%)	Recall (%)	Acc (%)	Params (M)
Haar	J = 1	95.82	95.38	96.26	98.61	49.80
	J = 2	96.20	95.72	96.68	98.75	49.95
	J = 3	96.45	95.98	96.92	98.82	50.21
	J = 4	96.36	95.88	96.82	98.78	50.63
DB4	J = 1	96.44	95.91	97.12	98.86	53.05
	J = 2	97.11	96.75	97.65	98.96	53.11
	J = 4	96.89	96.51	97.33	98.92	53.17
Ours (DB4)	J = 3	97.25	96.89	97.86	99.01	53.14

**Table 7:** Ablation Study on Gating Mechanisms. Fusion_RG indicates removing the interactive gating from the fusion module and directly passing the three input features to the Adaptive Weighting Fusion (AWF). Fusion_RA denotes removing the adaptive weights and computing a simple average of each modality’s information.

Methods	MaxF (%)	Pre (%)	Recall (%)	Acc (%)	Params (M)
Fusion_RG	96.35	96.18	96.94	98.65	48.33
Fusion_RA	96.40	95.92	96.88	98.78	52.87
Ours	97.25	96.89	97.86	99.01	53.14

## Data Availability

The dataset can be obtained from https://www.cvlibs.net/datasets/kitti/eval_road.php (accessed on 21 December 2025). Data are contained within the article.
